# Risk of New-Onset Long COVID Following Reinfection With Severe Acute Respiratory Syndrome Coronavirus 2: A Community-Based Cohort Study

**DOI:** 10.1093/ofid/ofad493

**Published:** 2023-10-05

**Authors:** Matthew L Bosworth, Boran Shenhuy, A Sarah Walker, Vahé Nafilyan, Nisreen A Alwan, Margaret E O’Hara, Daniel Ayoubkhani

**Affiliations:** Data and Analysis for Social Care and Health Division, Office for National Statistics, Newport, United Kingdom; Methodology and Quality Directorate, Office for National Statistics, Newport, United Kingdom; National Institute for Health Research Health Protection Research Unit in Healthcare-Associated Infections and Antimicrobial Resistance, University of Oxford, Oxford, United Kingdom; Nuffield Department of Medicine, University of Oxford, Oxford, United Kingdom; Data and Analysis for Social Care and Health Division, Office for National Statistics, Newport, United Kingdom; Faculty of Public Health, Environment and Society, London School of Hygiene and Tropical Medicine, London, United Kingdom; School of Primary Care, Population Sciences and Medical Education, Faculty of Medicine, University of Southampton, Southampton, United Kingdom; National Institute for Health Research Southampton Biomedical Research Centre, University of Southampton and University Hospital Southampton National Health Service Foundation Trust, Southampton, United Kingdom; Long Covid Support, Registered Charity, London, United Kingdom; Data and Analysis for Social Care and Health Division, Office for National Statistics, Newport, United Kingdom; Leicester Real World Evidence Unit, Diabetes Research Centre, University of Leicester, Leicester, United Kingdom

**Keywords:** coronavirus, COVID-19, long COVID, post-COVID condition, reinfection

## Abstract

**Background:**

Little is known about the risk of long COVID following reinfection with severe acute respiratory syndrome coronavirus 2 (SARS-CoV-2). We estimated the likelihood of new-onset, self-reported long COVID after a second SARS-CoV-2 infection, compared to a first infection.

**Methods:**

We included UK COVID-19 Infection Survey participants who tested positive for SARS-CoV-2 between 1 November 2021 and 8 October 2022. The primary outcome was self-reported long COVID 12–20 weeks after each infection. Separate analyses were performed for those <16 years and ≥16 years. We estimated adjusted odds ratios (aORs) for new-onset long COVID using logistic regression, comparing second to first infections, controlling for sociodemographic characteristics and calendar date of infection, plus vaccination status in participants ≥16 years of age.

**Results:**

Overall, long COVID was reported by those ≥16 years after 4.0% and 2.4% of first and second infections, respectively; the corresponding estimates among those aged <16 years were 1.0% and 0.6%. The aOR for long COVID after second compared to first infections was 0.72 (95% confidence interval [CI], .63–.81) for those ≥16 years and 0.93 (95% CI, .57–1.53) for those <16 years.

**Conclusions:**

The risk of new-onset long COVID after a second SARS-CoV-2 infection is lower than that after a first infection for persons aged ≥16 years, though there is no evidence of a difference in risk for those <16 years. However, there remains some risk of new-onset long COVID after a second infection, with around 1 in 40 of those aged ≥16 years and 1 in 165 of those <16 years reporting long COVID after a second infection.

## Teaser

In this community-based cohort study, the risk of new-onset long COVID was 28% lower after a second infection compared with a first infection in participants aged ≥16 years. There was no evidence of a difference in risk for those <16 years.

Long COVID describes symptoms such as fatigue, breathlessness, pain, and cognitive impairment that persist for months or years after a severe acute respiratory syndrome coronavirus 2 (SARS-CoV-2) infection and can affect a wide range of organ systems [1]. As of 2 January 2023, 2 million people in the United Kingdom (UK) (3.1% of the population) were estimated to be experiencing long COVID, with 1.5 million of these reporting limitations to their daily activities [2]. SARS-CoV-2 reinfection rates increased rapidly following the emergence of the Omicron variant and remain high. More than 90% of reinfections occurred during the period when the Omicron variants were dominant; as of 23 November 2022, the estimated rate of reinfection was 40.6 per 100 000 participant-days at risk, compared with 11.5 as of 13 December 2021 (before Omicron was the dominant variant) [3]. However, there is limited evidence regarding the risk of new-onset long COVID following SARS-CoV-2 reinfection.

Descriptive data from a survey administered by long COVID patient support groups in the UK suggest that most respondents with long COVID (89%) developed it after their first SARS-CoV-2 infection [4]. However, this finding is not generalizable to the whole population as the data were collected from social media support groups for people with long COVID (ie, a highly self-selecting group). Another study using data from electronic health records (EHRs) suggests that SARS-CoV-2 reinfection increases the risk of postacute sequelae such as death and organ-specific impairment up to 6 months postinfection [5]. However, the study sample of United States military veterans is unlikely to be representative of the broader population, and the study did not assess common long COVID symptoms.

We therefore investigated the risk of new-onset long COVID following a second SARS-CoV-2 infection and how this compares with first infections, using data from a large community-based sample selected at random from the UK population.

## METHODS

### Study Data and Design

The main data source for this analysis was the UK COVID-19 Infection Survey (CIS, ISRCTN21086382, https://www.ndm.ox.ac.uk/covid-19/covid-19-infection-survey/protocol-and-information-sheets), run by the Office for National Statistics and comprising a sample of more than half a million participants randomly selected from the UK community population (excluding communal establishments such as hospitals, care homes, halls of residence, and prisons). Households were invited to enroll in the survey between April 2020 and January 2022 (see Supplementary Table 1 for response rates). Data were collected via face-to-face interviews with trained study workers at participants’ home address until July 2022, when remote data collection was introduced. For most participants, this meant online data collection, but the option to respond via telephone was also available (for more information about the survey design, see [6]).

At each follow-up assessment, all participants answered a survey questionnaire including questions on confirmed/suspected SARS-CoV-2 infections and long COVID symptoms, and provided a nose-and-throat swab for polymerase chain reaction (PCR) testing. Blood testing was initially undertaken on consenting participants aged ≥16 years in a random 20% subsample of households from enrollment, as well as postenrollment from those in households where at least 1 household member had tested positive for SARS-CoV-2 on a swab. The serology subsample has been increased throughout the lifetime of the CIS, including expansion to children aged ≥8 years, and 55% of participants in this age group have now provided at least 1 blood sample [6].

CIS data for participants in England were linked to Pillar 1 (swab testing for SARS-CoV-2 in UK Health Security Agency laboratories and National Health Service hospitals for those with a clinical need, and health and care workers) and Pillar 2 (swab testing for SARS-CoV-2 in the wider population, through commercial partnerships, either processed in a laboratory or more rapidly via lateral flow device tests) SARS-CoV-2 test results [7]. To classify coronavirus disease 2019 (COVID-19) vaccination status and timing for participants in England, we used CIS responses linked to National Immunisation Management System records, with the latter being used when data conflicted. Vaccination information for participants in Wales, Scotland, and Northern Ireland was obtained from CIS responses alone.

### Inclusion and Exclusion Criteria

We included CIS participants who tested positive for SARS-CoV-2 using positive swab tests (PCR or lateral flow tests) obtained from national testing programs (participants in England) or during CIS follow-up (all participants), and self-reported positive swab tests (PCR or lateral flow tests) taken outside of the CIS.

**Figure 1.**
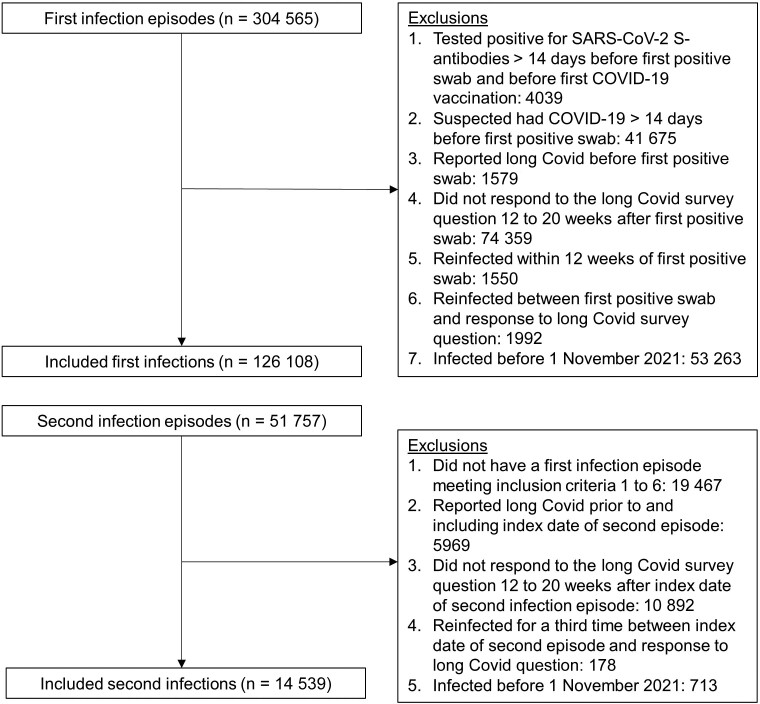
Study participant flow diagram. Inclusion criteria were applied sequentially. Abbreviations: COVID-19, coronavirus disease 2019; S, spike, SARS-CoV-2, severe acute respiratory syndrome coronavirus 2.

To identify first SARS-CoV-2 infection episodes (Figure 1), we excluded participants who reported suspected COVID-19 or tested positive for spike (S) antibodies (in the study or elsewhere [self-reported; very small number of individuals], ignoring blood tests taken after first COVID-19 vaccination) >2 weeks before their first positive swab; reported long COVID symptoms at any time before their first positive swab; did not respond to the survey question on long COVID 12 to 20 weeks after their first positive swab; or were reinfected within 12 weeks of their first positive swab or before their first response to the long COVID question 12 to 20 weeks after their first positive swab (since, if these participants experienced long COVID, it is uncertain whether their symptoms were attributable to the first or second infection).

To identify second SARS-CoV-2 infection episodes (Figure 1), we excluded participants with a second episode who did not have a first infection episode meeting the abovementioned criteria; reported long COVID prior to (and including) the start of their second episode; did not respond to the long COVID question 12 to 20 weeks after the start of their second episode; or were reinfected again before their first response to the long COVID question 12 to 20 weeks after the start of their second episode.

After identifying first and second infection episodes, in order to ensure a reasonable degree of overlap in the calendar date of first and second infection episodes, we then excluded from analysis any infections occurring before 1 November 2021. This means that any participants who were first infected before 1 November 2021 and were reinfected after this date were only included in the second infection group. This date was chosen based on distribution percentiles; the fifth percentile of the calendar date distribution was 6 December 2020 for first infections but 13 November 2021 for second infections among those ≥16 years of age; and 10 December 2020 and 20 October 2021, respectively, among those <16 years of age (Supplementary Figure 1).

### Exposure Definition

The exposure was a second versus a first SARS-CoV-2 infection, defined by adapting previous methods used for producing official statistics relating to SARS-CoV-2 surveillance in the UK [8, 9].

Positive swab test results from any source were grouped into infection episodes to allow for long duration of PCR positivity in some individuals, also incorporating information from genetic sequencing, S-gene target positivity, and cycle threshold (Ct) values, together with negative PCR test results from CIS only. We defined a new infection episode as a new swab positive occurring >120 days after an index positive with the preceding test being negative, or >90 days with the preceding 2 consecutive tests being negative (1 negative after 20 December 2021 when Omicron variants dominated given higher reinfection rates with Omicron), or >60 days with the 3 preceding consecutive tests being negative, or after 4 preceding consecutive negative test results at any time.

We further split these infection episodes if they contained multiple sequences from different genetic lineages (eg, BA.5 and BA.2), had incompatible S-gene target positivity with Ct <30 (eg, S-gene positive and S-gene negative, both with Ct <30), or had large decreases in Ct within a set of positive tests grouped together or low Ct long after the first positive within an episode (both indicative of a new infection rather than ongoing PCR positivity). We also split infection episodes where a new lateral flow device positive was recorded 27 days or more after the start of an infection episode, or 19 days or more after a previous positive PCR or lateral flow test, since this again indicates high viral load and actively replicating virus (more likely associated with a new infection).

### Outcome

The primary outcome was new-onset long COVID of any severity according to the survey question “Would you describe yourself as having long COVID, that is, you are still experiencing symptoms more than 4 weeks after you first had COVID-19, that are not explained by something else?” based on the National Institute for Health and Care Excellence (NICE) guidelines for defining the long-term effects of COVID-19 [10]. Participants who responded positively to this question were then also asked about the extent to which their symptoms limited their ability to undertake daily activities (“Does this reduce your ability to carry out day-to-day activities compared with the time before you had COVID-19?” Response options: “Yes, a lot”; “Yes, a little”; “Not at all”), and the presence or absence of 21 individual symptoms attributed to long COVID (the most commonly reported when the survey question was developed [11–13]). The secondary outcome was activity-limiting long COVID (no long COVID or long COVID without activity limitation vs activity limited a little or a lot by long COVID).

We considered participants’ first response to these questions 12 to 20 weeks after the date of the first positive swab in each infection episode (the index date). This definition of long COVID is consistent with the NICE and World Health Organization definitions of post–COVID-19 syndrome and post–COVID-19 condition, respectively [10, 14]. We included an 8-week window to allow for differences in scheduling of study visits (eg, visits being rescheduled to a later date than originally planned).

### Covariates

Covariates included sociodemographic characteristics (age, sex, White or non-White ethnicity [non-White ethnic groups were combined due to low numbers of participants reporting long COVID], area deprivation quintile group, and self-reported preexisting health conditions), vaccination status, mode of response to the survey at follow-up for long COVID (remote or face-to-face interview; to account for the increased likelihood of self-reported long COVID among those responding remotely [15]), calendar date of infection (to account for changes in dominant SARS-CoV-2 variant in circulation and other temporal effects), and the number of days from the index date for each infection episode to follow-up for long COVID.

Vaccination status was defined using a combination of the number of doses and time since last dose to account for vaccine waning. By the end of our study period in October 2022, all adults in the UK had been offered a primary vaccine course (a first plus a second dose) and at least 1 booster dose. In the spring of 2022, an additional booster dose was offered to adults aged ≥75 years, people in care homes, and those aged ≥12 years who were immunocompromised. Starting in September 2022, a further booster campaign was gradually rolled out for adults aged ≥50 years, care home residents and staff, frontline health and social care workers, people with caring responsibilities, and those who were clinically vulnerable or were household contacts of immunocompromised individuals [16].

### Statistical Methods

Separate analyses were conducted for participants aged ≥16 years and those aged <16 years. We compared study participants’ sociodemographic characteristics at the first and second infection using means for continuous variables and proportions for categorical variables, with absolute standardized differences ≥10% indicating a large imbalance between infection episodes [17].

We calculated the crude percentage of participants reporting long COVID 12 to 20 weeks after each infection episode to estimate the absolute risk of new-onset long COVID. We also calculated the prevalence of a range of long COVID symptoms as the percentage of those aged ≥16 years who reported having long COVID after each infection. This was not possible for participants <16 years of age due to small sample sizes.

Adjusted odds ratios (aORs) for long COVID 12 to 20 weeks postinfection were estimated from binary logistic regression models, comparing second infection episodes to first infection episodes (reference group). For those ≥16 years of age, models were adjusted for all the covariates outlined above. The models for those <16 years of age were adjusted for age, sex, calendar date of infection, and the number of days from the index date to long COVID follow-up due to an insufficient number of events for some levels of the other covariates. We did not adjust for COVID-19 vaccination status in those aged <16 years because of the high correlation with age and underlying health status; children aged <5 years are not eligible for vaccination in the UK, and uptake has been low among those aged 5–11 years (just 5.2% of the population of England in this age group had received 2 doses of a COVID-19 vaccine by 8 October 2022 [18]). All variables were defined at the index date of each infection episode except mode of response, which was defined at the date of the response to the long COVID question.

Continuous variables (age, follow-up time, and calendar date of infection) were modeled as restricted cubic splines, with boundary knots at the 10th and 90th percentiles and an internal knot at the median of the distributions. We tested 1 to 5 knots and selected 1 internal knot as this minimized the Bayesian information criterion for the models.

As it is possible that the impact of reinfection on the development of new-onset long COVID varies across different subpopulations, for the primary outcome, we used likelihood ratio tests to test for effect modification of the association between reinfection and new-onset long COVID, by interacting reinfection with each of the covariates included in the models.

All statistical analyses were performed using R version 3.6 software.

## RESULTS

### Description of the Study Sample

After applying the study inclusion and exclusion criteria (Figure 1), the analysis included 126 108 first infections (110 844 in those ≥16 years of age, 15 264 in those <16 years of age) and 14 539 second infections (11 244 in those aged ≥16 years, 3295 in those aged <16 years) occurring between 1 November 2021 and 8 October 2022 (Table 1). Median follow-up time from the start of infection to long COVID response was 102 days (interquartile range [IQR], 92–112) for those ≥16 years of age and 101 days (IQR, 92–111) for those <16 years of age.

**Table 1.** Characteristics of Study Participants

Characteristic^a^	Age ≥16 y at Infection			Age <16 y at Infection		
	First Infection (n = 110 844)	Second Infection (n = 11 244)	Absolute Standardized Difference, %	First Infection (n = 15 264)	Second Infection (n = 3295)	Absolute Standardized Difference, %
Age, y, mean (SD)	53.9 (16.6)	47.3 (15.9)	40.8	9.8 (3.4)	10.5 (3.0)	22.1
Calendar time of infection, No. of days since 1 November 2021, mean (SD)	144.8 (81.8)	189.8 (84.8)	54.0	91.8 (64.1)	165.5 (82.8)	99.5
No. of days from index date to long COVID follow-up, mean (SD)	103.1 (13.2)	103.1 (12.9)	0.3	102.8 (13.4)	103.2 (13.1)	3.5
Sex			5.1			0.2
Female	60 572 (54.6)	6431 (57.2)	…	7484 (49.0)	1613 (49.0)	…
Male	50 272 (45.4)	4813 (42.8)	…	7780 (51.0)	1682 (51.0)	…
Race/ethnicity			10.3			3.0
White	104 073 (93.9)	10 253 (91.1)	…	13 295 (87.1)	2836 (86.1)	…
Non-White	6771 (6.1)	991 (8.8)	…	1969 (12.9)	459 (13.9)	…
Area deprivation quintile group			10.7			4.9
1 (most deprived)	10 481 (9.5)	1261 (11.2)	…	1546 (10.1)	388 (11.8)	…
2	17 178 (15.5)	2023 (18.0)	…	2312 (15.1)	532 (16.1)	…
3	22 983 (20.7)	2303 (20.5)	…	3095 (20.3)	638 (19.4)	…
4	27 810 (25.1)	2644 (23.5)	…	3760 (24.6)	776 (23.6)	…
5 (least deprived)	32 392 (29.2)	3013 (26.8)	…	4551 (29.8)	961 (29.2)	…
Self-reported preexisting health conditions^b^			11.2			2.4
No	91 573 (82.6)	9742 (86.6)	…	14 291 (93.6)	3065 (93.0)	…
Yes	19 271 (17.4)	1502 (13.4)	…	973 (6.4)	230 (7.0)	…
Mode of response to survey			45.6			75.0
Face-to-face	66 987 (60.4)	4297 (38.2)	…	13 165 (86.2)	1783 (54.1)	…
Remote (online or telephone)	43 857 (39.6)	6947 (61.8)	…	2099 (13.8)	1512 (45.9)	…
Vaccination status^c^			54.7			20.0
Unvaccinated	1545 (1.4)	384 (3.4)	…	11 327 (74.2)	2309 (70.1)	…
1 dose ≥14 d previously	1197 (1.1)	195 (1.7)	…	2561 (16.8)	490 (14.9)	…
2 doses/booster dose ≥14–89 d previously	28 644 (25.8)	1998 (17.8)	…	632 (4.1)	182 (5.5)	…
2 doses/booster dose 90–179 d previously	44 634 (40.3)	3271 (29.1)	…	532 (3.5)	230 (7.0)	…
2 doses/booster dose 180–269 d previously	28 362 (25.6)	4035 (35.9)	…	…	…	…
2 doses/booster dose ≥270 d previously	6462 (5.8)	1361 (12.1)	…	212 (1.4)	84 (2.5)	…

Data are presented as No. (%) unless otherwise indicated.

Abbreviations: COVID, coronavirus disease; SD, standard deviation.

^a^All characteristics (except mode of response) were defined at index date for each infection episode.

^b^Obtained from the survey question “Do you have any physical or mental health conditions or illnesses lasting or expected to last 12 months or more, excluding any long-lasting COVID-19 symptoms?”

^c^Counts have been aggregated for those <16 years of age in the 2 doses/booster dose 180–269 days previously and ≥270 days previously due to small sample sizes. Standardized differences were calculated on the raw counts.

Of those ≥16 years of age in the first infection episode group, 40.3% had received 2 or more doses of a COVID-19 vaccine 90–179 days before infection. In the second infection episode group, 35.9% had received at least 2 doses of a COVID-19 vaccine 180–269 days before infection. Most of those <16 years of age were unvaccinated in both the first (74.2%) and second (70.1%) infection episode groups.

Among those ≥16 years of age, the mean age was higher for first infection episodes (53.9 years [standard deviation, 16.6 years]) than second infection episodes (47.3 years [standard deviation, 15.9 years]), and a larger percentage reported having a preexisting health condition at the first infection episode (17.4%) than the second infection episode (13.4%).

### Long COVID in Participants ≥16 Years of Age

Long COVID of any severity was reported by 4381 of those aged ≥16 years after a first infection (prevalence, 4.0% [95% confidence interval {CI}, 3.8%–4.1%]) and 274 (2.4% [95% CI, 2.2%–2.7%]) following a second infection. Activity-limiting long COVID was reported by 3103 of those aged ≥16 years (2.8% [95% CI, 2.7%–2.9%]) after a first infection, compared with 180 (1.6% [95% CI, 1.4%–1.9%]) after a second infection.

**Figure 2.**
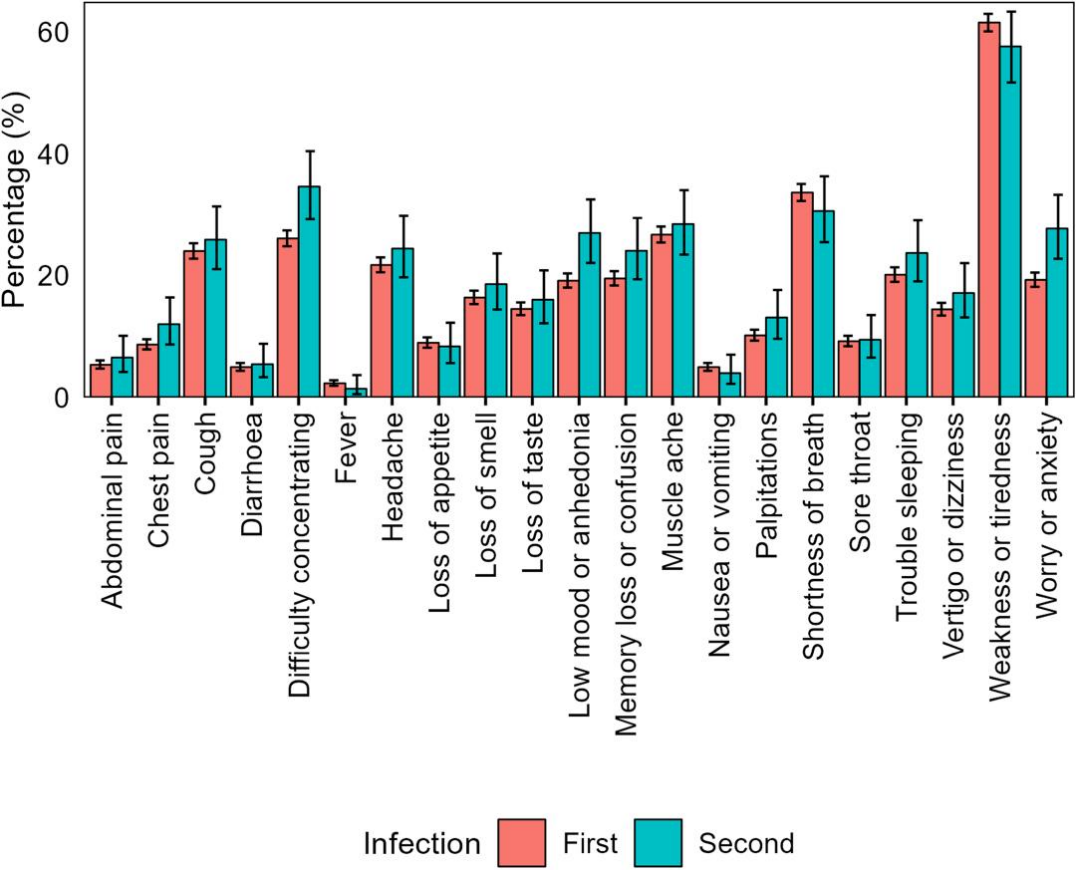
Prevalence of long COVID symptoms among participants ≥16 years of age who reported having long COVID after a first or second severe acute respiratory syndrome coronavirus 2 infection. Error bars represent 95% confidence intervals.

The most common symptoms among those aged ≥16 years with long COVID were fatigue (61.6% [95% CI, 60.1%–63.0%] after a first infection, 57.7% [95% CI, 51.8%–63.4%] after a second infection); shortness of breath (33.7% [95% CI, 32.3%–35.1%] and 30.7% [95% CI, 25.5%–36.4%], respectively); muscle ache (26.7% [95% CI, 25.4%–28.1%] and 28.5% [95% CI, 23.5%–34.1%], respectively), and difficulty concentrating (26.1% [95% CI, 24.8%–27.4%] and 34.7% [95% CI, 29.3%–40.5%], respectively) (Figure 2 and Supplementary Table 2). The prevalence of neuropsychological symptoms (eg, difficulty concentrating, memory loss or confusion, and worry or anxiety) was numerically higher following a second infection. However, the small number of participants reporting long COVID after a second infection (n = 274) prevented formal statistical testing.

**Figure 3.**
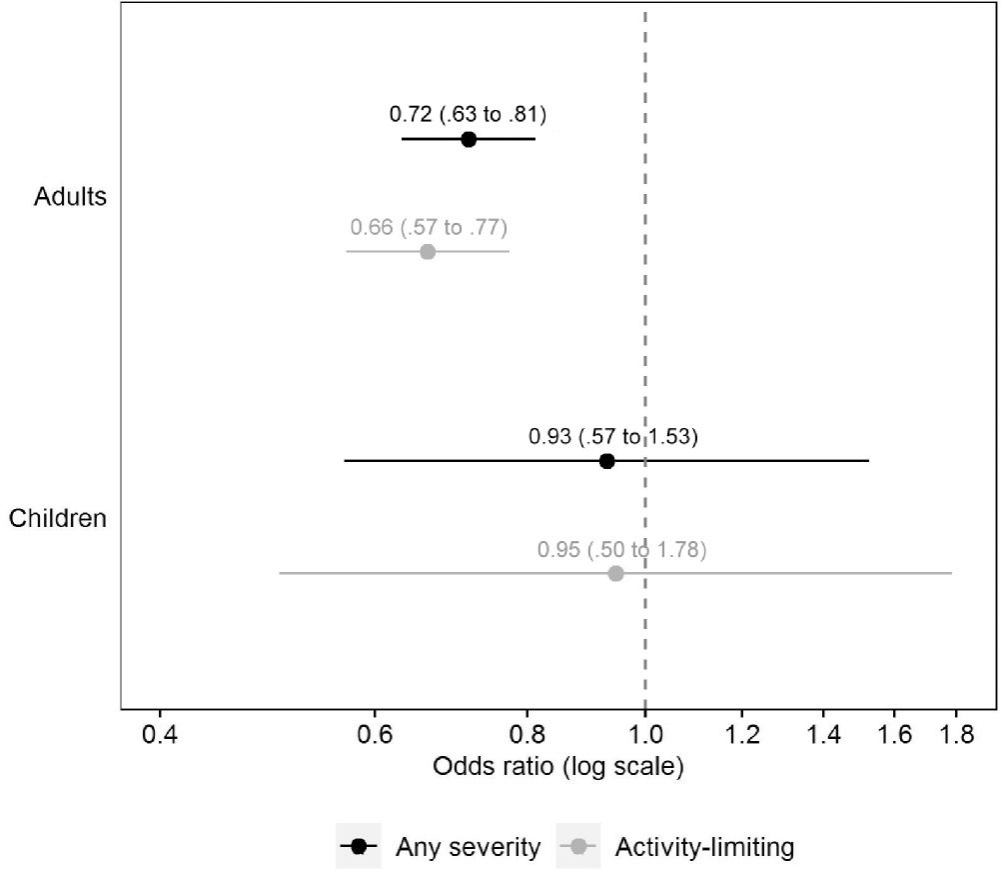
Adjusted odds ratios for long COVID 12 to 20 weeks after a second severe acute respiratory syndrome coronavirus 2 (SARS-CoV-2) infection compared with a first infection (reference group). Odds ratios for those ≥16 years of age are adjusted for sociodemographic characteristics (age, sex, White or non-White ethnicity, area deprivation quintile group, and self-reported health status), vaccination status, time from infection to follow-up for long COVID, calendar date of infection (as a proxy for the dominant SARS-CoV-2 variant in circulation), and mode of response to the survey. Odds ratios for those aged <16 years are adjusted for age, sex, time from infection to follow-up for long COVID, and calendar date of infection. Confidence intervals are at the 95% level.

The aOR of reporting long COVID after a second infection compared to a first infection was 0.72 (95% CI, .63–.81) for long COVID of any severity and 0.66 (95% CI, .57–.77) for activity-limiting long COVID (Figure 3). There was no evidence for effect modification of the association between reinfection and new-onset long COVID of any severity by age (*P* = .35), sex (*P* = .17), ethnicity (*P* = .98), area deprivation (*P* = .89), preexisting health status (*P* = .14), vaccination status (*P* = .15), or calendar date of infection (*P* = .29).

### Long COVID in Participants <16 Years of Age

Long COVID of any severity was reported by 160 of those aged <16 years after a first infection (prevalence, 1.0% [95% CI, .9%–1.2%]) and 20 (0.6% [95% CI, .4%–.9%]) following a second infection. Activity-limiting long COVID was reported by 87 of those <16 years of age (0.6% [95% CI, .5%–.7%]) after a first infection, compared with 12 (0.4% [95% CI, .2%–.6%]) after a second infection.

The aOR of reporting long COVID after a second infection compared to a first infection was 0.93 (95% CI, .57–1.53) for long COVID of any severity and 0.95 (95% CI, .50–1.78) for activity-limiting long COVID (Figure 3). There was no evidence for effect modification of the association between reinfection and new-onset long COVID of any severity by age (*P* = .78) or sex (*P* = .85). The interaction with calendar date of infection was statistically significant (*P* = .006). However, wide CIs meant there was a high degree of uncertainty around this finding, and the results should be interpreted with caution (Supplementary Figure 2).

## DISCUSSION

### Summary of Main Findings

Relative to a first SARS-CoV-2 infection, the odds of new-onset long COVID of any severity or activity-limiting long COVID were 28% and 34% lower, respectively, following a second infection in those ≥16 years, even after adjusting for vaccination status and other potential confounders. This finding may partly be the result of some degree of protection against long COVID being conferred by prior infection (assuming persistent symptoms were not present after the first infection), coupled with survivorship effects—that is, people with a greater predisposition to long COVID (eg, females or those with certain underlying health conditions [19]) experiencing persistent symptoms following a first infection, and therefore not being in the sample eligible to experience new-onset long COVID following a second infection.

In participants aged <16 years, the crude prevalence of new-onset long COVID was lower following a second infection compared with a first infection, but this difference was not statistically significant after controlling for confounders. However, CIs were wide, reflecting the smaller sample, and compatible with similar reductions to those seen in those ≥16 years of age.

### Comparison With Other Studies

Research into the risk of long COVID following reinfection with SARS-CoV-2 is scarce. Our findings are consistent with descriptive data from self-selecting respondents collected by long COVID patient support groups, which suggest that the majority of respondents who have long COVID developed it after their first infection [4]. However, most participants in the previous study were unvaccinated when they were first infected, and several studies have shown that being vaccinated is associated with a reduced risk of developing long COVID following SARS-CoV-2 infection [20–23]. Another study using EHRs found that reinfection increased the risk of postacute sequelae up to 6 months postinfection [5]. However, this study did not assess long COVID specifically, was based on a nonrepresentative sample of United States military veterans, and encompassed periods when the Alpha, Delta, and Omicron variants were the most common variants, whereas our study primarily covered the period when Omicron variants were predominant. Our analysis of a randomly selected community-based cohort shows that the risk of self-reported new-onset long COVID in those ≥16 years of age is lower following a second infection even after adjusting for vaccination status and calendar date of infection (as a proxy for the dominant SARS-CoV-2 variant in circulation at any given time).

Although the risk of new-onset long COVID in those ≥16 years of age was lower after a second SARS-CoV-2 infection than a first infection, the absolute risk is not negligible; 2.4%, that is around 1 in 40, of those ≥16 years who did not report long COVID after their first infection went on to do so after a second infection. Other evidence suggests that SARS-CoV-2 reinfection increases risk of postacute, multiorgan sequelae up to 6 months after reinfection, compared with a single infection [5]. Our study extends these findings by examining the relationship between reinfection and common long COVID symptoms. We found that most symptoms reported by those ≥16 years of age with new-onset long COVID after a second infection were reported at similar levels of prevalence by participants with long COVID after a first infection. There was some descriptive evidence that the prevalence of neuropsychological symptoms (eg, difficulty concentrating, memory loss or confusion, and worry or anxiety) was higher among participants reporting new-onset long COVID after a second infection, compared with those who reported it after a first infection. However, we were unable to adjust for characteristics associated with the likelihood of infection and the risk of developing long COVID in this analysis due to small event counts for many of the symptoms. Therefore, these results should be interpreted with caution as they may be driven by residual confounding.

The aim of our study was to estimate the risk of new-onset long COVID after reinfection, rather than the incremental risk conferred by reinfection in addition to that from the primary infection. Several studies have shown that previous infection with SARS-CoV-2 is associated with reduced risk of severe disease and hospital admission following reinfection, with the strongest association in those with hybrid immunity from vaccination and prior infection [24–26]. We found no evidence for effect modification of the association between reinfection and risk of new-onset long COVID by vaccination status, indicating lower odds of long COVID after a second infection compared to a first infection irrespective of vaccination status (although we note that this analysis may have been underpowered, and absence of evidence does not necessarily imply evidence of absence). Since the pathophysiology of long COVID is poorly understood [27], future research should investigate the biological mechanisms underlying the association between previous immunity and the reduction in risk of developing long COVID observed in this study. This could improve understanding of the pathogenesis of long COVID and potentially improve therapeutics.

### Strengths and Limitations

The main strength of the analysis is the use of data from CIS, comprising approximately half a million people randomly sampled from private households to minimize selection bias. CIS participants are routinely tested for SARS-CoV-2, so our study sample included infections that were initially asymptomatic as well as symptomatic infections. We adjusted for a wide range of factors that may be related to both the risk of reinfection [3] and developing long COVID [19, 21]. However, the observational nature of the study means that unmeasured confounding may remain, and thus causality cannot be inferred. In particular, we were only able to adjust for age, sex, calendar date, and follow-up time in the analysis of participants <16 years of age due to limited sample sizes. We adjusted for self-reported preexisting health status as a proxy for underlying health conditions, which is a good predictor of chronic health conditions derived from EHRs [28]. No data were available on whether participants received antiviral treatment during the acute phase of infection or other treatments for long COVID during follow-up.

The routine testing in CIS also means that we can more completely ascertain infection history compared with using results from national testing programs or self-report alone. We exploited multiple sources of information, including genetic sequencing, S-gene target positivity, and Ct values to distinguish as much as possible between persistent PCR positivity and new infections. However, 1 limitation is that inevitably some short infections and/or reinfections may have been missed.

We excluded participants who were reinfected <12 weeks after their first infection or before they had responded to the long COVID question 12 to 20 weeks after their first infection. Although only a small number of participants (n = 3542 [1.2% of the original sample of first infections]) were excluded for this reason, this could introduce bias if a shorter duration of first infection is related to the risk of long COVID. Consequently, the results may not be generalizable to people who are reinfected with short intervals between their first and second infection.

Another limitation is that long COVID status was self-reported, so outcome misclassification is possible. Some participants may have been experiencing symptoms because of a health condition unrelated to COVID-19 (including other respiratory viruses), while others who did have long COVID may not have described themselves as such (eg, due to the perceived stigma associated with the condition [29]). Conversely, self-recognition of long COVID (participants’ perception of the change in their own health compared with preinfection) may be more reliable than EHRs in some respects, for example due to differences in healthcare-seeking behaviors between sociodemographic groups and long COVID diagnoses being underrecorded in primary care [30].

Long COVID is a relapsing and remitting condition [11]. Since we only assessed long COVID at one study visit 12–20 weeks after each infection, this may mean that the prevalence of long COVID was underestimated in this study. Further work could explore how the long-term trajectories of long COVID and recovery rates compare after a first infection compared with subsequent infections.

This analysis only includes infections occurring between 1 November 2021 and 8 October 2022. The Omicron COVID-19 variant was first identified in the UK on 27 November 2021 [31] and quickly became the main variant in circulation. Most first and second infections in our sample are therefore Omicron infections, and it is unclear whether our findings are representative of infections with other SARS-CoV-2 variants. Reinfections became more common following the emergence of the Omicron variant [3], and the risk of long COVID has previously been shown to be lower for infections compatible with the Omicron variants compared with the Delta variant [32, 33]. However, it is important to note that the population prevalence of long COVID in the UK has remained relatively stable since the emergence of the Omicron variant due to higher infection rates compared with earlier periods in the pandemic [2].

## CONCLUSIONS

The risk of new-onset long COVID after a second SARS-CoV-2 infection is lower than that after a first infection for those ≥16 years of age even after adjusting for vaccination status and variant (using calendar date as a proxy). Although there was no statistical evidence of a difference in risk between first and second infections for those <16 years of age, there was a large degree of uncertainty around the point estimate, suggesting this finding could be a consequence of lower power in this smaller subgroup. Despite our finding that reinfection carries a lower risk of new-onset long COVID than a first infection in those ≥16 years of age, there remains some risk of new-onset long COVID, following about 1 in 40 second infections among those ≥16 years of age. Further research is required to understand whether the risk of long COVID is reduced with each subsequent infection. This is essential to model the expected future burden of long COVID on the population.

## Supplementary Material

ofad493_Supplementary_Data

## Supplementary Data

Supplementary materials are available at *Open Forum Infectious Diseases* online. Consisting of data provided by the authors to benefit the reader, the posted materials are not copyedited and are the sole responsibility of the authors, so questions or comments should be addressed to the corresponding author.

## Notes

***Author contributions.*** M. L. B., D. A., and B. S. conceptualized and designed the study. M. L. B. and B. S. prepared the study data and performed the statistical analysis. All authors contributed to interpretation of the results. M. L. B. and D. A. were responsible for the first draft of the manuscript. All authors contributed to critical revision of the manuscript and approved the final manuscript.

***Participant consent.*** Ethical approval was obtained from the South Central Berkshire B Research Ethics Committee (20/SC/0195). At enrollment, participants aged ≥16 years provided written consent, including for optional weekly follow-up assessments for 1 month followed by at least 12 monthly assessments for the majority of participants. Parents and carers provided consent on behalf of those aged 2–15 years, while those aged 10–15 years also provided written assent.

***Patient involvement.*** M. E. O. and N. A. A. have lived experience of long COVID.

***Data availability.*** De-identified study data are available to accredited researchers in the Office for National Statistics Secure Research Service under part 5, chapter 5 of the Digital Economy Act 2017. For further information about accreditation, contact research.support@ons.gov.uk or visit: https://www.ons.gov.uk/aboutus/whatwedo/statistics/requestingstatistics/approvedresearcherscheme.

***Disclaimer.*** The views expressed are those of the authors and are not necessarily those of the National Health Service, the National Institute for Health Research (NIHR), the Department of Health and Social Care, or the UK Health Security Agency (UKHSA). For the purpose of open access, the authors have applied a Creative Commons Attribution (CC BY) licence to any Author Accepted Manuscript version arising.

***Financial support.*** The CIS is funded by the Department of Health and Social Care and the UKHSA with in-kind support from the Welsh government; the Department of Health on behalf of the Northern Ireland government; and the Scottish government. There was no dedicated funding for this study of CIS data. D. A. is supported by the NIHR Applied Research Collaboration East Midlands. A. S. W. is supported by the NIHR Health Protection Research Unit in Healthcare-Associated Infections and Antimicrobial Resistance (NIHR200915), a partnership between the UKHSA and the University of Oxford. A. S. W. is also supported by the NIHR Oxford Biomedical Research Centre and is an NIHR Senior Investigator. N. A. A. is a co-investigator on NIHR-supported research on long COVID (STIMULATE-ICP and HI-COVE studies).
