# Supplementary material for: Risk of New-Onset Long COVID Following Reinfection With Severe Acute Respiratory Syndrome Coronavirus 2: A Community-Based Cohort Study
Source: Open Forum Infect Dis. 2023 Oct 5;10(11):ofad493. doi: 10.1093/ofid/ofad493 (PMC10633780; doi:10.1093/ofid/ofad493)
Supplement: ofad493_Supplementary_Data [file ofad493_supplementary_data.docx]

**Supplementary Materials**

**Supplementary Figure 1.** Density plots of calendar date of infection (before excluding infections occurring before 1 November 2021), stratified by infection episode and age group.


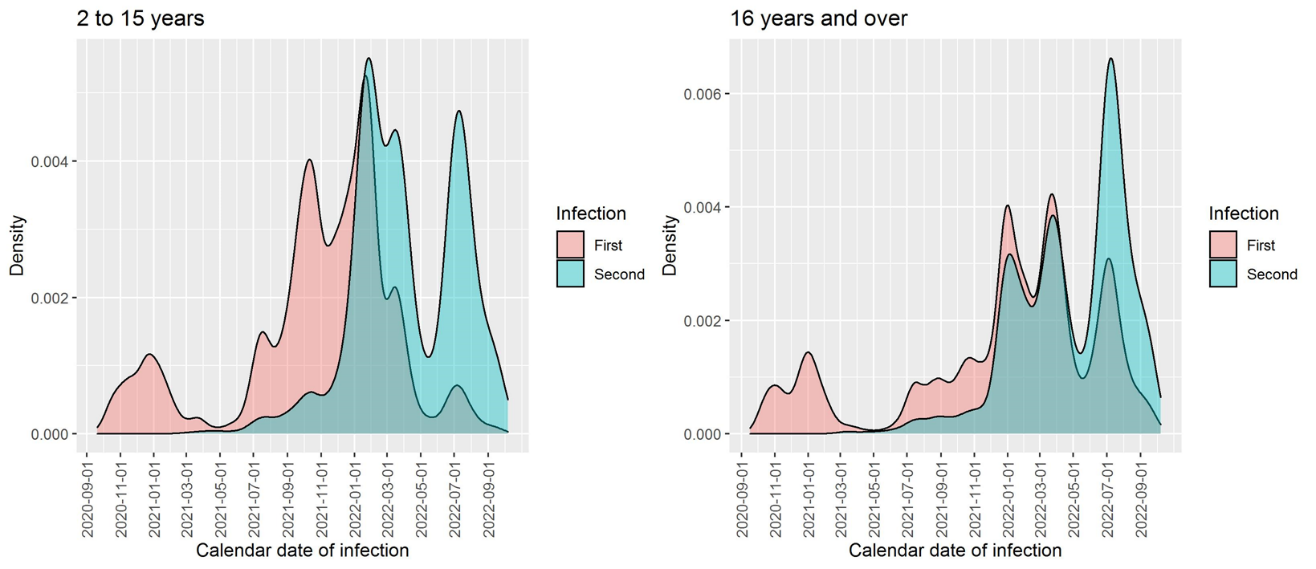


**Supplementary Figure 2.** Estimated marginal probability of Long Covid by calendar date of infection in those <16 years.^1^


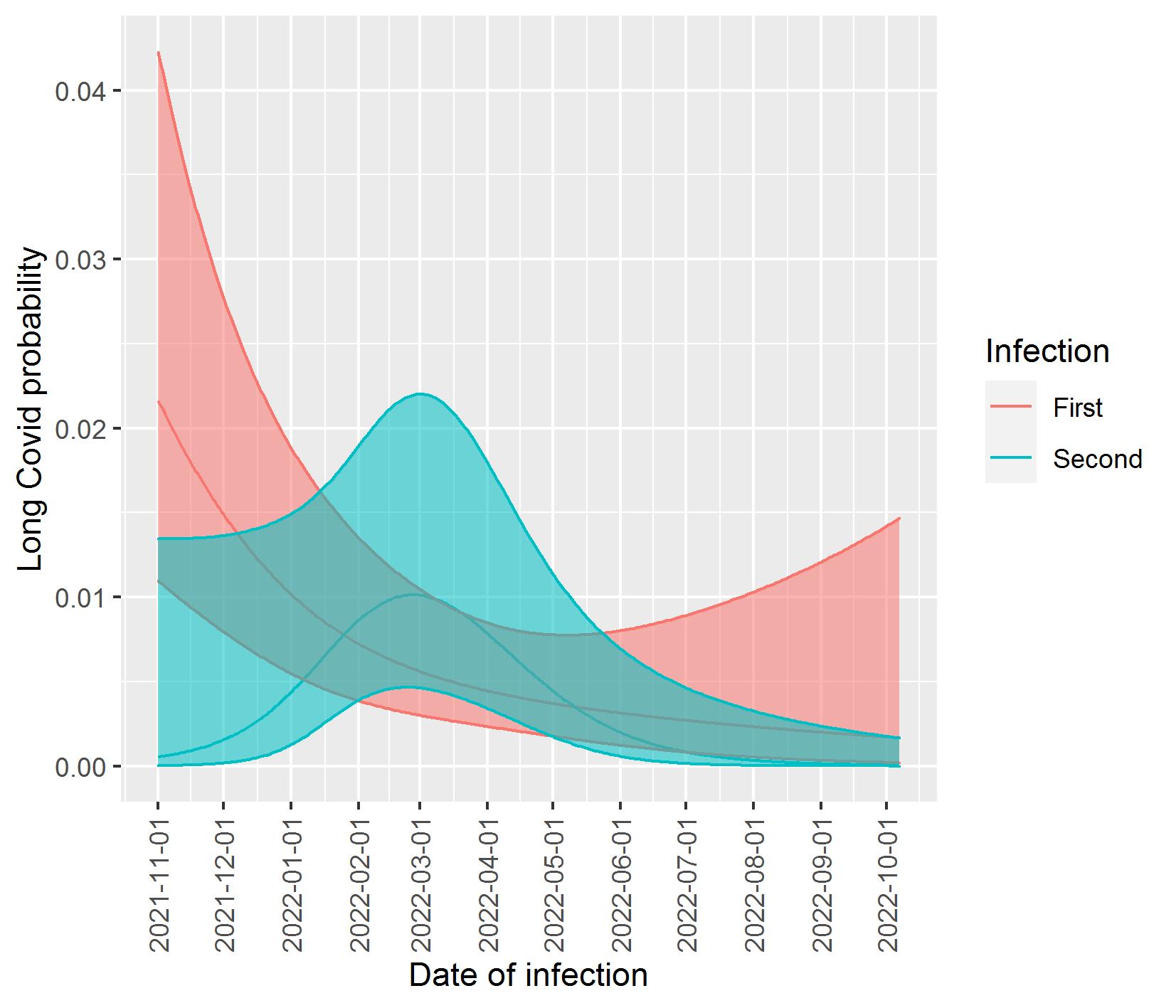


^1^ Estimates were calculated using the emmeans package, adjusting for age, sex, and time from infection to follow-up for Long Covid. Shaded areas are 95% confidence intervals.

**Supplementary Table 1.** Number and percentage of households invited to participate in the COVID-19 Infection Survey who subsequently enroled, by country and phase of study

| **Study phase** | **England** | **Wales** | **Northern Ireland** | **Scotland** |
| --- | --- | --- | --- | --- |
| Initial invitation | 10,266 (51%) | 7,031 (41%) | 7,373 (43%) | N/A |
| Extension period | 39,392 (43%) | N/A | N/A | N/A |
| AddressBase | 173,583 (12%) | 7,051 (14%) | N/A | 23,217 (13%) |

Notes: The initial invitation phase was open to previous respondents to ONS surveys who had consented to participate in future research, and started on 26 April 2020 in England, 29 June 2020 in Wales, and 26 July 2020 in Northern Ireland. The extension period refers to the period of time beyond the initial pilot phase of the study when the sample was increased, and started on 31 May 2020 in England. Sampling from AddressBase started on 13 July 2020 in England, 5 October 2020 in Wales, and 14 September 2020 in Scotland, and involved randomly selecting addresses from an address list. Enrolment rates are as of 31 January 2022, when recruitment into the study ended, and are taken from the technical dataset accompanying the official COVID-19 Infection Survey publication: https://www.ons.gov.uk/peoplepopulationandcommunity/healthandsocialcare/conditionsanddiseases/d atasets/covid19infectionsurveytechnicaldata

**Supplementary Table 2.** Percentage (%) of participants who reported each of the Long Covid symptoms among those >16 years who reported having Long Covid after a first or second SARS-CoV-2 infection, with 95% confidence intervals

| **Symptom** | **Long COVID after first infection**  **(N=4,381)** | **Long COVID after second infection**  **(N=274)** |
| --- | --- | --- |
| Abdominal pain | 5.4 (4.8 to 6.1) | 6.6 (4.2 to 10.1) |
| Chest pain | 8.7 (7.9 to 9.6) | 12.0 (8.7 to 16.4) |
| Cough | 24.0 (22.8 to 25.3) | 25.9 (21.1 to 31.4) |
| Diarrhoea | 5.0 (4.4 to 5.7) | 5.5 (3.4 to 8.8) |
| Difficulty concentrating | 26.1 (24.8 to 27.4) | 34.7 (29.3 to 40.5) |
| Fever | 2.4 (1.9 to 2.8) | 1.5 (0.6 to 3.7) |
| Headache | 21.8 (20.6 to 23.0) | 24.5 (19.7 to 29.9) |
| Loss of appetite | 9.0 (8.2 to 9.9) | 8.4 (5.7 to 12.3) |
| Loss of smell | 16.4 (15.3 to 17.5) | 18.6 (14.5 to 23.6) |
| Loss of taste | 14.5 (13.5 to 15.6) | 16.1 (12.2 to 20.9) |
| Low mood or not enjoying | 19.2 (18.0 to 20.4) | 27.0 (22.1 to 32.6) |
| Memory loss or confusion | 19.5 (18.4 to 20.7) | 24.1 (19.4 to 29.5) |
| Muscle ache | 26.7 (25.4 to 28.1) | 28.5 (23.5 to 34.1) |
| Nausea or vomiting | 5.0 (4.4 to 5.7) | 4.0 (2.3 to 7.0) |
| Palpitations | 10.2 (9.3 to 11.1) | 13.1 (9.6 to 17.7) |
| Shortness of breath | 33.7 (32.3 to 35.1) | 30.7 (25.5 to 36.4) |
| Sore throat | 9.2 (8.4 to 10.1) | 9.5 (6.6 to 13.5) |
| Trouble sleeping | 20.2 (19.0 to 21.4) | 23.7 (19.1 to 29.1) |
| Vertigo or dizziness | 14.5 (13.5 to 15.5) | 17.2 (13.2 to 22.1) |
| Weakness or tiredness | 61.6 (60.1 to 63.0) | 57.7 (51.8 to 63.4) |
| Worry or anxiety | 19.3 (18.2 to 20.5) | 27.7 (22.8 to 33.3) |
